# Comprehensive investigation of *CASK* mutations and other genetic etiologies in 41 patients with intellectual disability and microcephaly with pontine and cerebellar hypoplasia (MICPCH)

**DOI:** 10.1371/journal.pone.0181791

**Published:** 2017-08-07

**Authors:** Shin Hayashi, Daniela Tiaki Uehara, Kousuke Tanimoto, Seiji Mizuno, Yasutsugu Chinen, Shinobu Fukumura, Jun-ichi Takanashi, Hitoshi Osaka, Nobuhiko Okamoto, Johji Inazawa

**Affiliations:** 1 Department of Molecular Cytogenetics, Medical Research Institute and Graduate School of Medical and Dental Sciences, Tokyo Medical and Dental University, Tokyo, Japan; 2 Hard Tissue Genome Research Center, Tokyo Medical and Dental University, Tokyo, Japan; 3 Department of Neurobiology and Kavli Institute for Neuroscience, Yale University School of Medicine, New Haven, CT, United States of America; 4 Genome Laboratory, Medical Research Institute, Tokyo Medical and Dental University, Tokyo, Japan; 5 Department of Pediatrics, Central Hospital, Aichi Human Service Center, Kasugai, Japan; 6 Department of Pediatrics, University of the Ryukyus School of Medicine, Nishihara, Japan; 7 Department of Pediatrics, Sapporo Medical University School of Medicine, Sapporo, Japan; 8 Department of Pediatrics, Tokyo Women's Medical University Yachiyo Medical Center, Yachiyo, Japan; 9 Department of Pediatrics, Jichi Medical School, Tochigi, Japan; 10 Department of Medical Genetics, Osaka Women's and Children's Hospital, Osaka, Japan; 11 Bioresource Research Center, Tokyo Medical and Dental University, Tokyo, Japan; Centre National de la Recherche Scientifique, FRANCE

## Abstract

The *CASK* gene (Xp11.4) is highly expressed in the mammalian nervous system and plays several roles in neural development and synaptic function. Loss-of-function mutations of *CASK* are associated with intellectual disability and microcephaly with pontine and cerebellar hypoplasia (MICPCH), especially in females. Here, we present a comprehensive investigation of 41 MICPCH patients, analyzed by mutational search of *CASK* and screening of candidate genes using an SNP array, targeted resequencing and whole-exome sequencing (WES). In total, we identified causative or candidate genomic aberrations in 37 of the 41 cases (90.2%). *CASK* aberrations including a rare mosaic mutation in a male patient, were found in 32 cases, and a mutation in *ITPR1*, another known gene in which mutations are causative for MICPCH, was found in one case. We also found aberrations involving genes other than *CASK*, such as *HDAC2*, *MARCKS*, and possibly *HS3ST5*, which may be associated with MICPCH. Moreover, the targeted resequencing screening detected heterozygous variants in *RELN* in two cases, of uncertain pathogenicity, and WES analysis suggested that concurrent mutations of both *DYNC1H1* and *DCTN1* in one case could lead to MICPCH. Our results not only identified the etiology of MICPCH in nearly all the investigated patients but also suggest that MICPCH is a genetically heterogeneous condition, in which *CASK* inactivating mutations appear to account for the majority of cases.

## Introduction

The *CASK* gene (OMIM: *300172), which encodes a member of the MAGUK (membrane-associated guanylate kinase) protein family, is highly expressed in the mammalian nervous system of both adults and fetuses, and plays several roles in neural development and synaptic functions [1, 2]. Since 2008, when both a heterozygous deletion and point mutations affecting *CASK* were reported to cause intellectual disability and microcephaly with pontine and cerebellar hypoplasia (MICPCH, OMIM: #300749) [3, 4], various types of *CASK* aberrations have been reported in more than 50 MICPCH patients [5, 6]. Due to its location on the X chromosome, loss-of-function of *CASK* usually leads to the manifestation of MICPCH in females, whereas a complete loss of this gene is believed to be lethal in males. Indeed, the vast majority of typical MICPCH by *CASK* aberrations are seen in female patients [4–8].

Since our first report [3], we have recruited 41 MICPCH patients to investigate their etiologies by genetic testing of *CASK* and other genome-wide approaches such as single nucleotide polymorphism (SNP) array, targeted resequencing and whole-exome sequencing. Besides 32 cases in which the molecular diagnosis could be attributed to *CASK* loss-of-function mutations, including a rare somatic mosaic mutation in a male patient, we identified a mutation in *ITPR1*, another gene in which mutations are causative for MICPCH. Moreover, in the analysis we identified aberrations in genes that might be associated with MICPCH: *HDAC2*, *MARCKS*, *RELN*, and possibly *HS3ST5*. Interestingly, our findings also suggested that concurrent mutations of both *DYNC1H1* and *DCTN1* might cause MICPCH. Our current study comprehensively clarified the etiologies of MICPCH in the cohort of patients and suggested heterogeneity of MICPCH.

## Results

Overall, we identified causative or candidate genomic aberrations in 37 of the 41 patients (90.2%) (Table 1). Briefly, 23 patients had point mutations of *CASK*, nine patients had CNVs affecting *CASK* and five had aberrations involving other genes. Among all, parental samples were available for 13 patients (Table 1). Their samples were also tested to confirm that all the patients’ mutations were *de novo*, except for patient 37.

**Table 1:** Clinical features and analysis of the 41 MICPCH patients.

Patient	Gender	Age	OFC (SD)		Developmental delay	Muscular hypotonia	Seizures	Other clinical features^a^	Gene(s) in which mutation was found	Mutation		Inher-itance^c^	Previous report
			At birth	Present						Description	Detection^b^		
1	F	2y8m	-3.2	-4.3	Severe	+	-		*CASK*	c.79C>T (p.R27*)	DS	*NA*	Patient 1 in [7]
2	F	1y5m	-1.2	-3.6	Severe	+	-		*CASK*	c.79C>T (p.R27*)	DS	*NA*	
3	F	2y0m	-2.3	-3.5	Moderate	+	-	Bilateral hydronephrosis	*CASK*	c.316C>T (p.R106*)	DS	*NA*	Patient 2 in [7]
4	F	4y3m		-9.2	Very severe		+		*CASK*	c.316C>T (p.R106*)	DS	*NA*	Patient 3 in [9]
5	F	1y		-3.6	Severe	-	-	VE	*CASK*	c.868G>T (p.E290*)	DS	*NA*	
6	F	2y8m	-2.8	-4.0	Severe	-	-	Severe hyperopia	*CASK*	c.2632C>T (p.Q878*)	DS	dn	Patient 3 in [7]
7	F	11m	-0.8	-3.2	Severe	-	-		*CASK*	c.243_244delTA (p.Y81*)	DS	*NA*	Patient 4 in [7]
8	F	5m	-0.9	-4.5	*Present*^d^	+	-		*CASK*	c.761_762delCT (p.S246*)	DS	dn	
9	F	15y		-6.4	Severe	+	+		*CASK*	c.1006_1012delACCTCCT (p.T336Qfs*23)	DS	*NA*	
10	F	4y2m		-4.0	Severe				*CASK*	c.2103delT (p.F701Lfs*26)	DS	*NA*	
11	F	1y	-1.4	-6.0	Severe	-	-	Large tongue	*CASK*	c.1677dupG (p.R560Afs*20)	WES	dn	
12	F	11y		-6.8	Severe		+	Hypertonia, scoliosis	*CASK*	c.1896dupC (p.C633Lfs*2)	DS	*NA*	
13	F	17y	-1	-6.0	Severe		+		*CASK*	c.2508delT (p.L837*)	DS	*NA*	
14	F	7y	-0.3	-4.0	Severe				*CASK*	c.173_173+1delGG	DS	*NA*	Patient 2 in [9]
15	F	7y9m	-3.4	-4.5	Severe	-	-		*CASK*	c.357-1G>A	DS	dn	Patient 5 in [7]
16	F	1y		-4.8	Moderate				*CASK*	c.1582+G>A	DS	*NA*	
17	F	14y	-0.4	-6.0	Moderate		-		*CASK*	c.2040-1G>C	DS	dn	Patient 6 in [7]
18	F	3y		-3.0					*CASK*	c.2302+1G>T	DS	*NA*	
19	F	11y		-5.0	Moderate				*CASK*	c.2302+1delT	DS	*NA*	Patient 1 in [9]
20	F	8y		-3.0	Severe				*CASK*	c.1910G>A (p.G637D)	DS	*NA*	Patient 4 in [9]
21	M	2y	-1	-4.1	Severe				*CASK*	c.1061T>C (p.L354P)	DS	*NA*	Patient 16 in [9]
22	M	4y4m		-4.9					*CASK*	c.317G>C (p.R106P)	DS	dn	
23	M	2y	-1.1	-3.2	Severe	+	+		*CASK*	c. [= /1493_1503+10delATGAACCAATGGTAAGTAGGAinsGG] (p.D498Gfs*12)	DS	*NA*	
24	F	5y	-3.1	-6.0	Severe	-	-	Squint with nystagmus and myopia, minor anomalies^e^	*CASK*	arr Xp11.4p11.3(41,500,243–45,480,187)x1	MA	dn	Patient in [3]
25	F	1y9m	-4.3	-4.6	Severe	+	-	Bilateral sensorineuronal deafness	*CASK*	arr Xp11.4p11.3(41,009,876–44,100,501)x1	MA	dn	Patient 7 in [7]
26	F	6y4m	-0.8	-3.2	Severe		+	Preterm birth at 33 weeks	*CASK*	arr Xp11.4p11.3(41,618,898–43,755,475)x1	MA	dn	
27	F	2y0m	-1.3	-4.0	Moderate	-	-		*CASK*	arr Xp11.4p11.3(41,337,795–42,468,013)x1	MA	dn	Patient 8 in [7]
28	F	4y	-2	-4.0					*CASK*	arr Xp11.4p11.3(41,145,925–46,090,321)x1	MA	*NA*	
29	F	12y8m	-4.8	-8.0	Severe			Glaucoma, PHPV	*CASK*	arr Xp11.4p11.3(41,163,139–44,592,980)x1	MA	*NA*	
30	F	12y	-1.9	-5.4	Severe		+	Severe scoliosis, strabismus	*CASK*	arr Xp11.4(41,405,593–41,570,391)x3	MA	*NA*	Patient 9 in [7]
31	F	7y2m	-1.5	-5.2	Severe	+	+	Internal strabismus	*CASK*	arr Xp11.4(41,382,179–41,540,922)x3 arr Xp11.22(56,012,908–56,275,153)x3	MA	dn	Patient 10 in [7]
32	F		0	-4.4	Severe				*CASK*	arr Xp11.4(41,442,660–41,527,850)x3	MA	*NA*	
33	F	10y	-0.9	-3.5	Severe	-	+	Hypoplastic CC, minor anomalies^f^	*HDAC2*, *MARCKS*	arr 6q21q22.31(109,497,085–122,505,593)x1	MA	*NA*	
34	F	2y		-4.0	Severe		+	Hirsutism, characteristic face	*RELN*	c.4918A>G (p.I1640V)	TR	*NA*	
35	M	4y6m		-5.0	Severe			Hypoplastic CC	*RELN*	c.7093G>A (p.V2365M)	TR	*NA*	
36	M	3y	0.2	-1.7	Severe			Thick CC	*ITPR1*	c.7753A>C (p.T2585P)	WES	dn	
37	F			-0.8	Moderate	+	-	VSD, minor anomalies^h^	*DYNC1H1*, *DCTN1*	*DYNC1H1* c.11824C>T (p.P3942S), *DCTN1* c.497C>G (p.S166C)	WES	*pat*,*mat*^g^	
38	F		-0.6	-5.2	Severe	+	+	Internal strabismus, VE	*ni*				
39	F	4y	-1	-3.1	Moderate			Hyperopia	*ni*				
40	F	5y	normal	-3.0	Severe				*ni*				
41	F	5y		0.6									

SD: standard deviation, ni: not identified

a PHPV: persistent hyperplastic primary vitreous CC: corpus callosum VE: ventricular enlargement

b DS: direct sequencing WES: whole exome sequencing MA: microarray TR: target resequencing

c dn: *de novo* m: maternal NA: Not available

d The severity has not been estimated correctly because of patient's age.

e Hirsutism, low hairline, arching of eyebrows with sparse lateral third, earlobe sinuses, micrognathia, proximally placed thumbs, brachydactyly and clinodactyly of the 5^th^ fingers

f Widely spaced eyes, downslanted palpebral fissure, Epicanthus, thick and small auricle, short philtrum, cubitus varus, short finger, proximal placement of thumb

g DCTN1 mutation was paternally and DYNC1H1 mutation was maternally inherited, respectively.

h Bilateral. preaxial polysyndactyly of toes, prominent forehead, depressed nasal root

### Subjects

**Fig 1:**
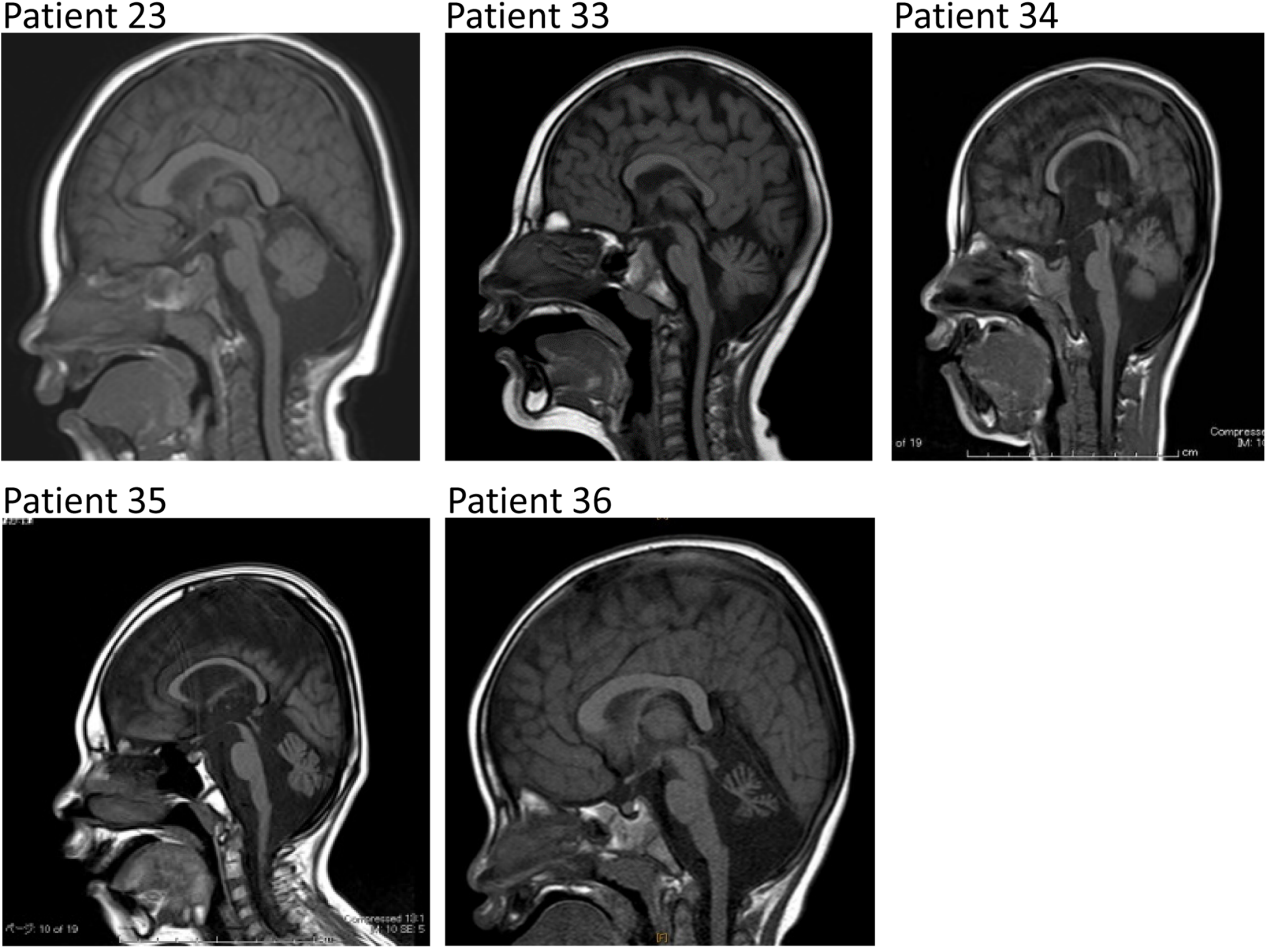
Representative sagittal images of brain MRI scans of five patients. Regardless of the distinct etiologies, all patients show features resembling MICPCH.

We recruited 41 patients clinically diagnosed with MICPCH, including 16 patients who have previously been reported [3, 7, 9] (Table 1). All patients were examined and evaluated by clinical dysmorphologists in each hospital and were enrolled in this study if they met the criteria for both microcephaly and pontocerebellar hypoplasia in brain magnetic resonance imaging (MRI) (Fig 1). All the patients were Japanese.

Patient’s clinical features are shown in Table 1. The cohort was largely comprised of females, with only six male patients, and their ages varied between five months to 17 years old at the last follow-up examination. Development has been markedly retarded, and some of them also showed seizures. Only six patients could walk at the time of the evaluation, and only three could speak a few words. While the occipital-frontal circumference (OFC) at birth ranged from normal to severe microcephaly in a few cases, at the last examination most of the patients showed a severe microcephaly (<-3.0SD). Although three cases (patients 36, 37, and 41) had OFC larger than -3.0 SD, we decided to include them in this cohort because their pontocerebellar hypoplasia resembled those of the other patients. Although most patients did not show major anomalies besides microcephaly, some of them also presented with ocular abnormalities and/or muscular hypotonia. From the results of the brain MRI examination, the severity of the pontocerebellar hypoplasia was found to be variable, whereas the size of the corpus callosum tended to be normal [10].

DNA and chromosomes were extracted from peripheral blood by standard methods. A lymphoblastoid cell line (LCL) was also established for all patients and available samples of parents (Table 1) by infecting lymphocytes with an Epstein-Barr virus, as previously described [11].

### Point mutation of *CASK*

The sequencing of the whole coding region of *CASK* identified point mutations likely to be responsible for the phenotypes in a total of 23 cases (Table 1). Nonsense mutations were detected in six cases: c.79C>T (p.R27*) in patients 1 and 2; c.316C>T (p.R106*) in patients 3 and 4; c.868G>T (p.E290*) and c.2632C>T (p.Q878*) in patients 5 and 6, respectively. Indel mutations introducing a premature stop codon were detected in seven cases: c.243_244delTA (p.Y81*), c.761_762delCT (p.S246*), c.1006_1012delACCTCCT (p.T336Qfs*23), c.2103delT (p.F701Lfs*26), c.1677dupG (p.R560Afs*20), c.1896dupC (p.C633Lfs*2), and c.2508delT (p.L837*) in patients 7 to 13, respectively. Specifically, the mutation in patient 11 was actually identified by WES, as described later. Mutations at the exon-intron junction were found in six cases: c.173_173+1delGG, c.357-1G>A, c.1582+G>A, c.2040-1G>C, c.2302+1G>T, and c.2302+1delT in patients 14 to 19, respectively. These mutations probably resulted in aberrant splicing, of which we have previously confirmed the presence of aberrant transcripts in patients 15 and 17 [7].

Missense mutations were detected in three cases, and *in silico* analysis predicted them to be disease-associated variations. The c.1910G>A (p.G637D) variant in patient 20, c.1061T>C (p.L354P) variant in patient 21, and c.317G>C (p.R106P) variant in patient 22 were predicted *in silico* to be damaging or disease-associated variation by PolyPhen-2, SIFT, or SNPs&GO, respectively, and their REVEL scores were also high (S1 Table).

**Fig 2:**
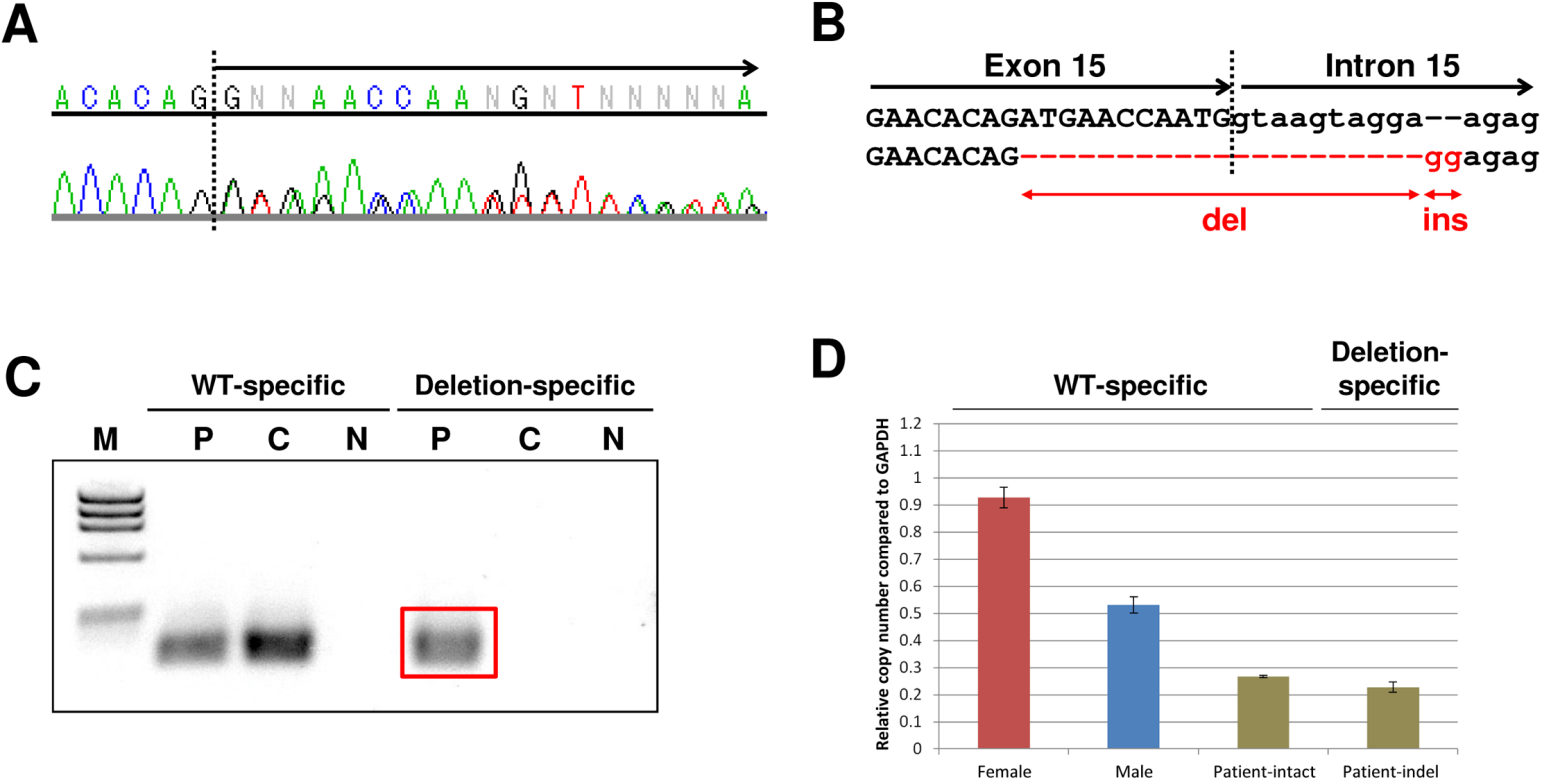
Detailed analysis of the mosaicism of *CASK* in patient 23. **A** Sequence chromatogram showing a heterozygous-like pattern in the latter part of exon 15 (arrow). **B** Scheme of the indel mutation. Compared with the reference allele (upper), the affected allele (lower) had a 21-bp deletion and a 2-bp insertion at the exon-intron junction of exon 15 and intron 15. **C** Results of the genomic PCR using WT-specific and indel-specific primer sets in the patient and a male control. The red box indicates a product amplified only in the patient with the indel-specific primer sets. M: marker; phiX174 RF DNA/Hae III Fragments, P: patient 23, C: control, N: negative control, no DNA added. **D** Real-time quantitative PCR of genomic DNA from patient 23 and male and female controls. While the relative copy number of the male control is naturally approximately half of that of the female control, those amplified with both WT-specific and deletion-specific primers in the patient are also approximately half of that of the male control.

Among the six male patients, *CASK* aberrations were identified in three; other than patients 21 and 22 described above, Patient 23 who presented with typical MICPCH (Fig 1) was found to have an indel between exon 15 and intron 15 resulting in a stop codon in a heterozygous-like pattern, despite being male (2A and 2B). This result suggests that the patient had the mutation in a mosaic state. Primer sets specific for the indel mutation amplified a product only in this patient (Fig 2C). Additionally, real-time quantitative PCR with the same primer sets confirmed a decreased copy number of both intact and indel alleles relative to a male control (Fig 2D). Thus, we concluded this patient is a rare case of a somatic mosaic indel mutation presumably affecting CASK expression, described as c. [= /1493_1503+10delATGAACCAATGGTAAGTAGGAinsGG].

### CNV involving *CASK* and other candidate genes

SNP array led to the discovery of heterozygous deletions involving whole or part of the *CASK* gene in six cases (patients 24 to 29), and intragenic duplications in three cases (patients 30 to 32) (Table 1). We have previously described patients 30 and 31 in detail [7]. In patient 32, oligonucleotide array determined exons 4 and 5 to be duplicated (S1 Fig). In order to precisely map the rearrangement, genomic PCR was performed with a primer combination specific to a tandem duplication structure (S2 Fig), and a product was detected only in the patient (S3 Fig). Sequencing of this product showed that intron 5 was followed by a duplicated intron 4 (S4 Fig), resulting in a shift in the reading frame and thereby producing an aberrant transcript. As a consequence, all intragenic duplications probably also resulted in loss-of-function of *CASK*.

Fine mapping of the CNVs using primer sets flanking the breakpoint (BP) junctions was performed in seven cases, patients 24–27 and 30–32. One case, patient 25, had a 126-bp sized *Alu* repeat at the BP (S5 Fig, upper), indicating it was likely mediated by non-allelic homologous recombination (NAHR) [12, 13]. In contrast, one case, patient 26, had no homology at the BP (S5 Fig, middle), therefore the rearrangement might have been induced by non-homologous end joining (NHEJ). The other five cases, patients 24, 27, and 30–32, had a microhomology of 1–3 base pairs at the BP junction (S5 Fig, lower), likely caused by microhomology-mediated break-induced replication (MMBIR). These findings suggest that most of the CNVs were not induced by a specific motif, suggesting CNVs involving *CASK* are probably non-recurrent (Table 1).

**Fig 3:**
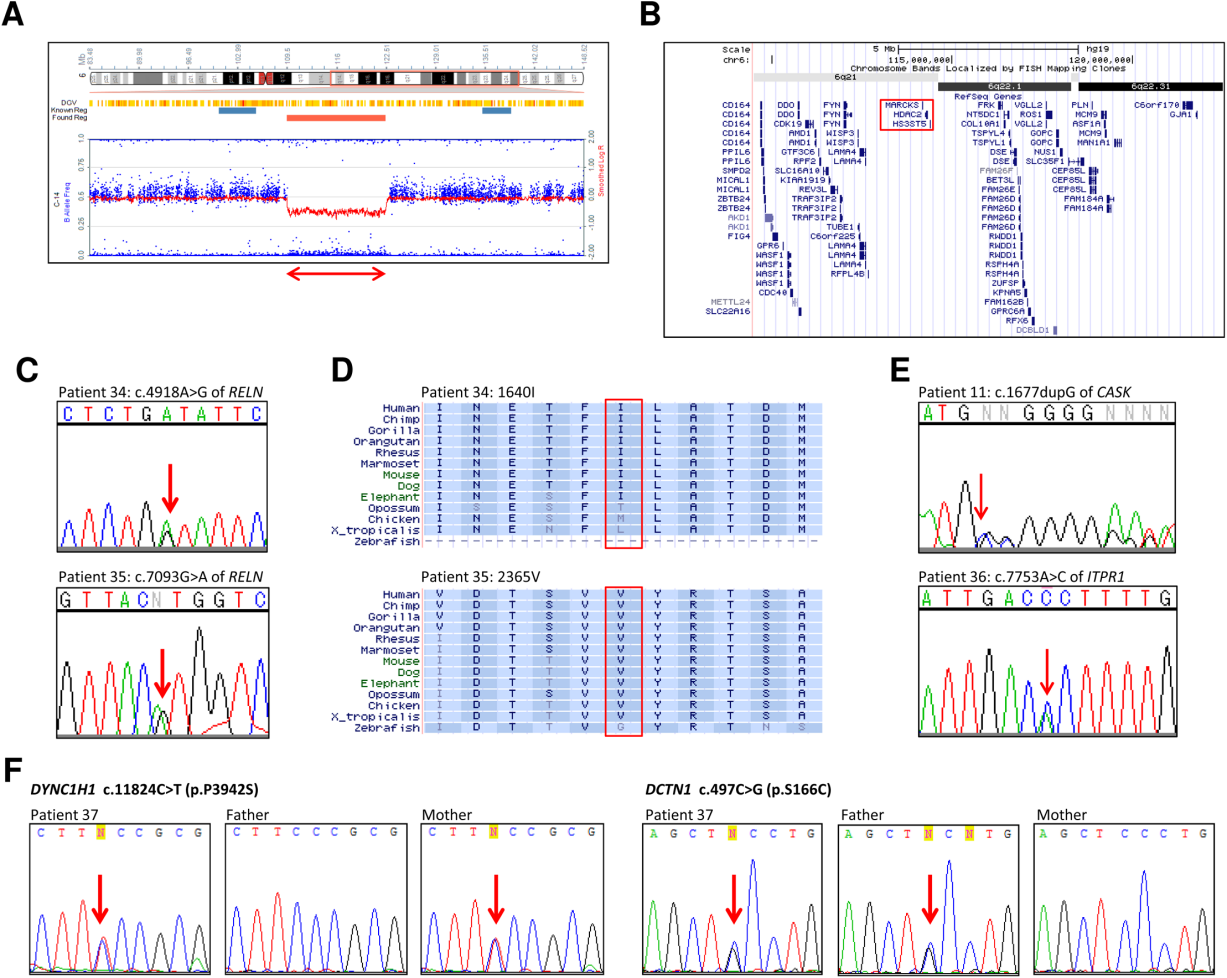
Genomic analysis of candidate genes other than *CASK*. (A) Result of the SNP array in patient 33 showing Heterozygous deletion at 6q21-q22.31 including *HDAC2* and *MARCKS*. This result is described as follows: arr 6q21q22.31(109,497,085–122,505,593)x1. The double-headed arrow indicates the deletion. (B) Mapping of the heterozygous deletion in patient 33. The red box denotes *HDAC2* and *MARCKS*. (C) Electropherograms depicting the mutations of *RELN* detected by targeted resequencing. Arrows indicate the mutated nucleotides. (upper) c.4918A>G (p.I1640V) in patient 34, (lower) c.7093G>A (p.V2365M) in patient 35. (D) Conservation of amino acids around each mutation of *RELN* in patient 34 (upper) and patient 35 (lower). The red box denotes the amino acid substituted by the mutation. (E) Electropherograms depicting the mutations detected by whole exome sequencing. Each arrow indicates the mutated nucleotide. (upper) c.1677dupG (p.R560Afs*20) of *CASK* in patient 11, (lower) c.7753A>C (p.T2585P) of *ITPR1* in patient 36. (F) Electropherograms depicting the mutations of *DYNC1H1* and *DCTN1* in patient 37 and her parents. Arrows indicate the mutated nucleotides. The left three panels indicating c.11824C>T (p.P3942S) of *DYNC1H1* show that the mutation is inherited from the mother, and the right three panels indicating c.497C>G (p.S166C) of *DCTN1* show the mutation is inherited from the father.

The SNP array analysis also identified a CNV not encompassing *CASK* in patient 33, who presented with typical MICPCH (Fig 1): a 13-Mb heterozygous deletion at 6q21-q22.31 (Table 1; Fig 3A and 3B). This result is concordant with previous reports of deletion overlapping with this case also showing MICPCH [14, 15]. We confirmed she had no mutations of *CASK* (data not shown).

### Targeted resequencing

Among 231 mutations detected in the six cases, only two mutations were in exons of *RELN* [OMIM: *600514] (S1 Table). One was c.4918A>G (p.I1640V) in patient 34, who is female and shows typical MICPCH (Fig 1), and the other was c.7093G>A (p.V2365M) in patient 35, who is male and also shows typical MICPCH (Fig 1). Both of the mutations were confirmed by direct sequencing (Fig 3C). The substituted isoleucine and valine are highly conserved at least in primates and placental animals (Fig 3D), suggesting that both amino acids are important for *RELN* function. *In-silico* analyses found p.I1640V not to be damaging (‘tolerated’ by PolyPhen-2 and SIFT, and ‘neutral’ by SNPs&GO), and scores of REVEL and CADD also suggested that mutation was not probably damaging (S2 Table). However, the mutated residue was predicted to be smaller than the wild type (WT), leading to loss of interactions, according to prediction by HOPE. The evaluation of p.V2365M may be vague: it was evaluated to be ‘damaging’ by PolyPhen-2 and SIFT and CADD scores were very high, while SNPs&GO judged it ‘neutral’ and REVEL score was rather low. HOPE predicted the 3D structure to be bigger than the WT and the mutated residue might prefer to be in another secondary structure, thus the local conformation would be slightly destabilized.

### Whole-exome sequencing

In patient 11 eight single-nucleotide variations (SNVs) and four indel mutations were extracted as candidate mutations. Among them, one insertion of a single guanine, c.1677dupG (p.R560Afs*20) in exon 18 of *CASK* caused a frameshift leading to a stop codon (Fig 3E). As the insertion was located at continuous guanines and close to the exon-intron junction, it might have been overlooked in the previous steps by the direct sequencing and targeted resequencing.

In patient 36, candidate SNVs were identified in three genes, *ITPR1*, *WNK3*, and *CYCL1*. Among them, c.7753A>C (p.T2585P) in exon 58 of *ITPR1* [OMIM: *147265] was functionally the strongest candidate (Fig 3E). Heterozygous mutations of *ITPR1* are known to cause spinocerebellar ataxia (SCA) 15 [#606658] and 29 [#117360]. Specifically, SCA29 is characterized by a psychomotor delay with an onset in the infantile period and an atrophic cerebellum, but not severe microcephaly [16], which is consistent with the mild microcephaly shown by patient 36 (Table 1, Fig 1). In total, *in*-*silico* analyses suggested this variant is probably disease-associated (S2 Table), and HOPE predicted that the mutation introduces a more hydrophobic residue at this position, hence the 3D structure of the protein might disrupt an α-helix, impairing the correct folding. Taken together, these findings indicate that this mutation may account for the patient’s etiology.

In patient 37, since the *de novo* analysis and compound heterozygous variant filtering failed to indicate a functionally significant candidate gene, we performed a protein-protein network analysis in order to assess possible interactions between the mutated genes found in the patient. Thus, all novel SNVs extracted in patient 37 were evaluated by the STRING database (http://string-db.org/), which indicated a close interaction between *DYNC1H1* [OMIM: *600112] and *DCTN1* [*601143], both being missense variants (Fig 3F, S6 Fig). While the maternally-inherited c.11824C>T (p.P3942S) variant in *DYNC1H1* was predicted *in silico* as benign to neutral with lower REVEL score, the paternally-inherited c.497C>G (p.S166C) mutation in *DCTN1* was predicted to be neutral to damaging (S2 Table). Despite the fact that the *in-silico* predictions did not show strong evidence of pathogenicity of the variants alone, the possibility remains that the combined occurrence of both variants in the same protein complex might be associated with the patient’s phenotype.

## Discussion

**Fig 4:**
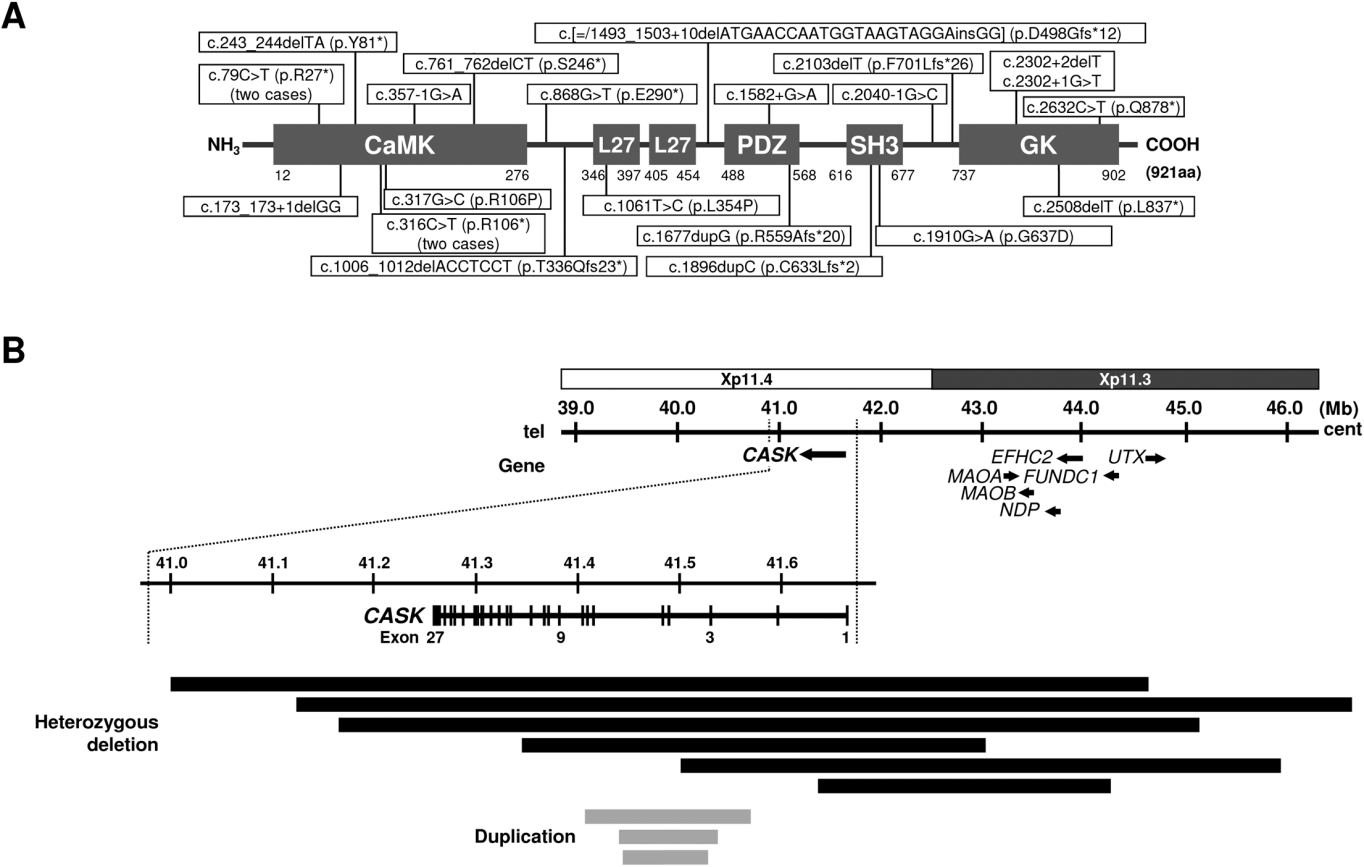
Schemes of the point mutations and CNVs involving CASK. (A) Schematic representation of the structure of *CASK* domains (NCBI Reference Sequence: NP_003679.2) and the position of the point mutations in patients 1–23. CaMK: calmodulin-dependent kinase, L27: LIN-2 and LIN-7 interaction, PDZ: PSD-95-Dlg-ZO1, SH3: Src homologous 3, GK: guanylate kinase. (B) Mapping of the CNVs involving *CASK* identified in patients 24–32. Black horizontal bars indicate the deletions and gray bars indicate the duplications, respectively, and horizontal arrows indicate genes and their directions. Dashed lines enlarge around *CASK*. The regional information is from the UCSC built on February 2009 (GRCh37/hg19).

Since the first report of *CASK* aberrations associated with MICPCH, more than 50 cases have been reported [4–7]. Here we summarized our investigation of 41 MICPCH cases, and in total we were able to identify causative or candidate genomic aberrations in 37 out of 41 cases (90.2%) (Table 1). Among the 37 positive cases, 23 cases (57.6%) had a point mutation of *CASK* and nine cases (27.3%) had CNV involving *CASK* (Table 1, Fig 4A and 4B). We also found five cases, two males and three females, with aberrations in other causative and/or candidate genes. For 13 cases whose parental samples were available, analysis of the parental samples showed that all the mutations were *de novo*, except patient 37 who may have compound mutations from both of the parents (see below).

Mapping of the breakpoint junctions of seven cases with the CNVs including CASK revealed that they might have been originated by several mechanisms of genomic rearrangements, namely NAHR, NHEJ and MMBIR (S2A–S2C Fig), suggesting that CNVs involving *CASK* are generally non-recurrent. This non-recurrent nature of *CASK* CNVs is concordant with the fact that most *CASK*-related point mutations arise *de novo*.

Atypical cases in this cohort were represented by three male cases found with *CASK* aberrations. Two cases (patient 21 and 22) had a missense mutation [9], and the other case (patient 23) was a somatic mosaic of WT and indel alleles (Fig 2). Recent studies have shown that *CASK* mutations in male patients lead to a broad spectrum of phenotypes including MICPCH. While missense mutations corresponding to functional change of the *CASK* protein causes non-syndromic intellectual disability [17–19] or FG syndrome [20], a decrease in the normal expression appears to correlate with the manifestation of MICPCH [8]. The complete loss of *CASK* expression was associated with a severe MICPCH phenotype and likely caused the reduced viability or *in utero* lethality [4, 8], which is consistent with the neonatal lethality of *Cask* knockout mice [21]. Thus, *CASK*-inactivating mutations in mosaic state (like the case of patient 23) might be able to escape early lethality due to incomplete loss of *CASK* expression, thereby resulting in typical MICPCH.

Aside from *CASK*, we also found another known gene in which mutations are causative for MICPCH, through the identification of an *ITPR1* mutation in patient 36 by WES (Fig 3E). *ITPR1* has previously been established as a causative gene of SCA29 associated with psychomotor delay and atrophy of the cerebellum, likely consistent with the brain malformation of patient 36 [16]. However, it may be distinguished from typical MICPCH caused by CASK mutation by a relatively milder microcephaly, thicker corpus callosum and non-atrophic brainstem.

Our investigation found a few genes that might be associated with MICPCH. Our findings in patient 33 support evidence linking the deletion of 6q21 to MICPCH (Fig 3A and 3B), concordant with previous reports of typical MICPCH cases with deletion at 6q21 [14, 15]. In particular, Case 7 reported by Rosenfeld showed typical MICPCH and had the smallest deletion, including only three protein-coding genes, *MARCKS* [OMIM: *602940], *HDAC2* [*605164], and *HS3ST5* [*609407]. MARCKS is reported to bind actin to support cytoskeletons, and HDAC2 is a histone deacetylase that acts as a suppressor of the expression of other genes [22]. While its function remains uncertain, HS3ST5 is highly expressed in the fetal brain [23]. These findings strongly suggest that haploinsufficiency of *HDAC2*, *MARCKS*, and possibly *HS3ST5* may be relevant to MICPCH, even though *Hdac2* or *Marcks* mutant mice showed conflicting phenotypes. *Marcks*^+/-^ mice showed no brain abnormality, while *Marcks*^-/-^ resulted in perinatal lethality with abnormal brain development [24]. Both heterozygous and homozygous *Hdac2*-deficient mice were viable and had normal brains, and additionally, the spine density was markedly increased in *Hdac2*^-/-^ mice [25]. On the other hand, mice with conditional knockout of both *Hdac2* and *Hdac1*, a homologous gene of *Hdac2*, showed severe hippocampal and cerebellar abnormalities [26]. Although these results suggested that either *HDAC2* or *MARCKS* alone might not cause brain malformations, haploinsufficiency of both genes may be relevant to MICPCH due to the collapse of the interaction between them.

Targeted resequencing screening only detected heterozygous mutations in *RELN* in two cases, of uncertain pathogenicity (Fig 3C). *RELN* encodes a secreted protein playing an important role in neural migration in the developing brain and spinal cord [27, 28]. While homozygous loss-of-function mutations of *RELN* cause lissencephaly with cerebellar hypoplasia [OMIM: #257320] [29, 30], heterozygous missense mutations of *RELN* cause autosomal-dominant lateral temporal epilepsy (ADLTE) with no brain anomaly [31]. Although the nature of the current mutations remain uncertain, an interesting report that CASK regulates the expression of RELN through interactions with TBR1 may suggest that *RELN* would be also associated with MICPCH [32].

WES analysis also identified an interesting pair of mutations of *DYNC1H1* and *DCTN1* in patient 37 (Fig 3F), respectively inherited from the mother and father. *DYNC1H1* encodes a member of the cytoplasmic dynein heavy chain [33], while *DCTN1* encodes p150(Glued), the largest subunit of dynactin [34, 35]. The dynein-dynactin complex plays an important role during mitosis and is necessary for synapse stabilization [36]. Heterozygous mutations in the motor domain of *DYNC1H1* have been associated with autosomal dominant mental retardation-13 (MRD13) with neuronal migration defects [OMIM: #614563], in which a subset of patients also present with microcephaly along with a small cerebellum and/or brainstem [37, 38]. However, the vast majority of the reported mutations were *de novo*. Mutations in *DCTN1* cause distal hereditary motor neuronopathy type VIIB [OMIM: *607641] and, so far, there is no association with brain malformations. Despite the uncertainty of the clinical significance of both variants, one might speculate the possibility of a ‘double-hit’ effect similar to a digenic inheritance model, in which variant genotypes at two loci (often involving interacting proteins) explain the phenotypes of some patients more clearly than the genotypes at one locus alone [39]. This tentative etiology may also explain the reason why the phenotype of patient 37 is relatively milder (Table 1). Nevertheless, further studies are needed to assess the combined effect of the two variants in the stability of the dynein-dynactin complex.

Our present study comprehensively explained the etiologies of MICPCH using several genomic analysis techniques. Not only did it identify *CASK* but also other genes involved in the etiology of MICPCH. Our findings demonstrate that MICPCH is a genetically heterogeneous condition, in which CASK inactivating mutations appear to account for the majority of MICPCH cases and with severer phenotypes, while the non-CASK mutation cases tend to have milder microcephaly. For the remaining cases, WES or WGS analysis may identify the undiscovered causative genes.

## Materials and methods

### Informed consent

Written informed consent was obtained from the parents of all the subjects. The consent form includes an agreement to have their clinical details and genetic analyses published without personal information.

### Direct sequencing of *CASK*

Point mutations within the coding region of *CASK* were analyzed by exon amplification and direct sequencing as previously described [7].

### Microarray analysis

For patients in whom no pathogenic *CASK* point mutation was found, SNP array (HumanOmniExpress-12 v1.0; Illumina, San Diego, CA, USA) was performed in order to detect genomic copy number variant (CNV) involving *CASK*. Oligonucleotide array (Human CGH Array 2.1Mb; Roche-Nimblegen, Madison, WI, USA) was subsequently applied to the positive cases to better determine the extent of the CNVs. DNA labeling, hybridization and washing were carried out according to the directions provided by the manufacturer. All CNVs were confirmed by fluorescence *in situ* hybridization (FISH), performed as previously described [40].

### Genomic PCR and real-time quantitative PCR

Genomic PCR was performed to amplify and sequence the breakpoint junctions of the CNVs. We also performed genomic PCR to confirm the presence of mosaicism in patient 19 by designing intact-specific and mutation-specific primer sets (S3 Table). The same primer sets were used for real-time quantitative PCR assay in order to determine the proportion of mosaic alleles on the genomic DNA of the patient, using the 7500 Real-Time PCR System (Applied Biosystems, Grand Island, NY, USA) and KAPA SYBR® FAST qPCR Master Mix (KAPA Biosystems, Wilmington, MA, USA), according to the manufacturers’ instructions.

### Targeted resequencing

Samples of six patients (patients 11, 34, 35, 38, 39, and 41) in whom neither point mutation of *CASK* nor CNVs involving *CASK* had been identified in the previous steps were subjected to targeted resequencing (amplicon sequencing) on the Ion Torrent PGM platform (Life Technologies, Carlsbad, CA, USA). Primer sets targeting the whole *CASK* region, including all exons including 5’ and 3’ untranslated regions (UTR), introns, promoter region, and all exons of 16 candidate genes interacting with CASK or corresponding to pontocerebellar hypoplasia in the literature (Table 2), were designed using the manufacturer’s AmpliSeq primer design tool (http://www.ampliseq.com). Library preparation and sequencing were performed according to the manufacturer's protocol. Base-calling and alignment to the human reference genome (GRCh37/hg19) were carried out using Torrent Suite 3.6 (Life Technologies). Variant detection was performed with the Variant Caller plug-in software of Torrent Suite.

**Table 2:** Candidate genes for the target resequencing.

Gene	Position	Exons	Target	Corresponding disorder (s)	Inheritance	Comment	Reference
*CASK*	Xp11.4	27	whole gene	MICPCH	XD	Causative gene	
*HDAC2*	6q21	14	all exons	MICPCH	AD	Candidate gene	
*TBR1*	2q24.2	6	all exons			Coactivator of CASK	[32]
*RELN*	7q22.1	65	all exons	Lissencephaly	AR	Causative gene	[30]
*VLDLR*	9p24.2	19	all exons	CAMRQ1	AR	Causative gene; a part of RELN signaling pathway	[41]
*DAB1*	1p32.2	15	all exons			Component of RELN signaling pathway	
*LRP8*	1p32.3	19	all exons			Component of RELN signaling pathway	
*FYN*	6q21	11	all exons			Component of RELN signaling pathway	
*VRK1*	14q32.2	12	all exons	PCH1A	AR	Causative gene	[42]
*EXOSC3*	9p13.2	4	all exons	PCH1B	AR	Causative gene	[43]
*TSEN54*	17q25.1	11	all exons	PCH2A, PCH4	AR	Causative gene	[44]
*TSEN2*	3p25.3	11	all exons	PCH2B	AR	Causative gene	[44]
*TSEN34*	19q13.42	5	all exons	PCH2C	AR	Causative gene	[44]
*SEPSECS*	4p15.2	11	all exons	PCH2D	AR	Causative gene	[45]
*RARS2*	1p32.3	1	all exons	PCH6	AR	Causative gene	[46]
*CHMP1A*	16q24.3	6	all exons	PCH8	AR	Causative gene	[47]
*BMI1*	10p12.2	9	all exons			Regulated by CHMP1A	[47]

PCH: pontocerebellar hypoplasia CAMRQ1: cerebellar ataxia, mental retardation, and dysequilibrium syndrome

AD: autosomal dominant AR: autosomal recessive XD: X-linked dominant

### Whole-exome sequencing

Among the eight negative cases after the targeted resequencing, we applied whole-exome sequencing (WES) for three cases (patients 11, 36, and 37) whose parental samples were available. Exome library was prepared using the SureSelect Human All Exon V5+UTRs Kit (Agilent, Santa Clara, CA, USA) and sequenced using the HiSeq2500 platform with Cluster/SBS kit v3 (Illumina). We extracted candidate mutations as follows: *de novo* variations were first extracted by comparing the sequence data between the trio of patient and parents, followed by filtering of variants with low read-depth (RD<4) or those occurring at non-coding regions. Next, variations observed in healthy populations were excluded in reference to a database of known SNPs, dbSNP [http://www.ncbi.nlm.nih.gov/SNP/], ExAC browser [http://exac.broadinstitute.org/], and Human Genetic Variation Database (HGVD), which provides Japanese genomic variants [http://www.genome.med.kyoto-u.ac.jp/SnpDB/]. The narrowed-down candidate mutations were confirmed by direct sequencing.

### Bioinformatics analysis of sequence variants

For *in-silico* prediction of a possible deleterious effect of missense mutations, we used the following online tools: Polymorphism Phenotyping v2 (PolyPhen-2) (http://genetics.bwh.harvard.edu/pph2), Sorting Intolerant from Tolerant (SIFT) (http://sift.jcvi.org/), SNPs&GO (http://snps.biofold.org/snps-and-go/), REVEL [48], CADD (http://cadd.gs.washington.edu/home), and HOPE (http://www.cmbi.ru.nl/hope/). The STRING database (http://string-db.org/) was used to assess the presence of functional interactions between proteins.

### Ethics statement

This study was approved by the Research Ethics Committee of Tokyo Medical and Dental University and all institutions involved in this project; The Ethics Committee of Central Hospital, Aichi Human Service Center, The Ethics Committee of the Ryukyus Graduate School of Medicine, Institutional Review Board of Kameda Medical Center, Institutional Review Board of Kameda Medical Center, Ethical Committee of Kanagawa Children Medical Center, The Institutional Review Board of Hokkaido Medical Center for Child Health and Rehabilitation, and The Ethical Review Board of Osaka Medical Center and Research Institute for Maternal and Child Health.

## Supporting information

S1 Fig Result of the oligonucleotide array of patient 32. An 85.19 kb duplication at Xp11.4 was detected (red circle). The result is described as follows: arr Xp11.4(41,442,660–41,527,850)x3.
(TIF)

S2 Fig Scheme of the expected tandem duplication in patient 32. The Box with a number indicate each exon. The pair of black triangles indicate the designed primer set for the genomic PCR.
(TIF)

S3 Fig Genomic PCR using the duplication-specific primer sets in patient 32. The arrow denotes the duplication-specific product. M: marker; phiX174 RF DNA/Hae III Fragments, P: patient 32, C: control, N: negative control, no DNA added.
(TIF)

S4 Fig Sequences around the breakpoint of the duplication of patient 32. P: sequence around the breakpoint of the patient, Ref-4 and Ref-5: reference sequences of a part of introns 4 and 5, respectively.
(TIF)

S5 Fig Schematic illustrations showing mechanisms producing the breakpoints of patient 21, 22, and 23. The size of each component doesn’t reflect the original proportion. (Upper) Two blue boxes denote *Alu* repeats flanking the deletion in patient 21. In the sequence of both proximal and distal *Alu* repeats, 118 of 126 bp (93.7%) are homologous, which likely induced the non-allelic homologous recombination (NAHR), resulting in the deletion. (Middle) There was no homology around the BP in patient 22 and the deletion might have been induced incidentally, while 10-bp sequences of unknown origin were observed at the BP. (Lower) Two yellow boxes denote 3-bp microhomologies, which likely induced the deletion in patient 23.
(TIF)

S6 Fig Scheme of protein interactions according to STRING. The WES analysis identified mutations including nonsense, splice-site, frameshift, and missense mutations, in those genes in patient 37. The enlarged panel shows a direct interaction between DYNC1H1 and DCTN1.
(TIF)

S1 Table Detected mutations in the target resequencing. (TIF)

S2 Table Scoring and judgements of *in silico* analyses. (TIF)

S3 Table Primer sets for detection of the mosaicism. (TIF)
