# Supplementary figures and images for: Comprehensive investigation of CASK mutations and other genetic etiologies in 41 patients with intellectual disability and microcephaly with pontine and cerebellar hypoplasia (MICPCH)
Source: PLoS One. 2017 Aug 7;12(8):e0181791. doi: 10.1371/journal.pone.0181791 (PMC5546575; doi:10.1371/journal.pone.0181791)

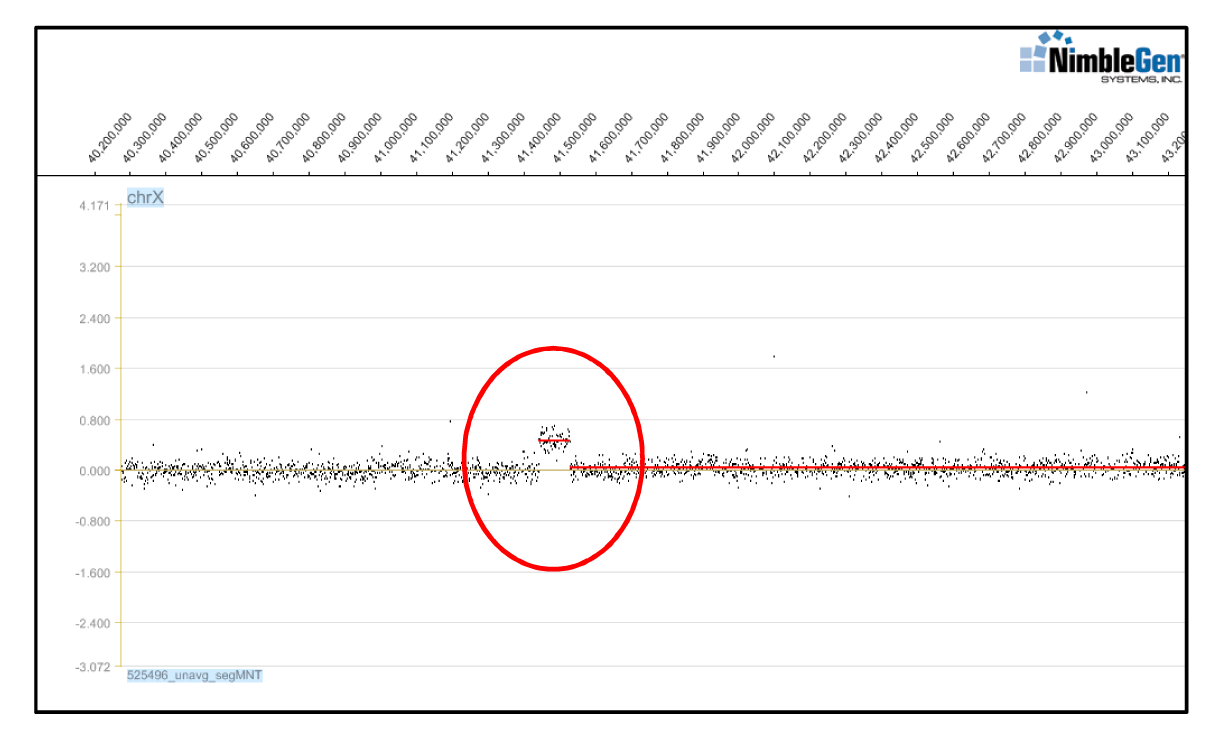

Supplement: S1 Fig — An 85.19 kb duplication at Xp11.4 was detected (red circle). The result is described as follows: arr Xp11.4(41,442,660–41,527,850)x3. (TIF) [file pone.0181791.s001.tif]

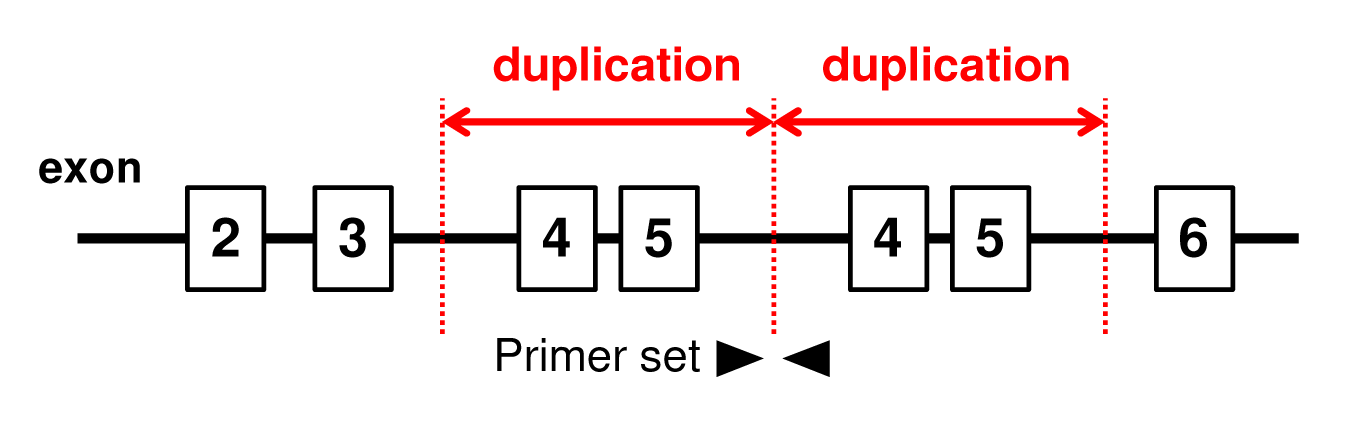

Supplement: S2 Fig — The Box with a number indicate each exon. The pair of black triangles indicate the designed primer set for the genomic PCR. (TIF) [file pone.0181791.s002.tif]

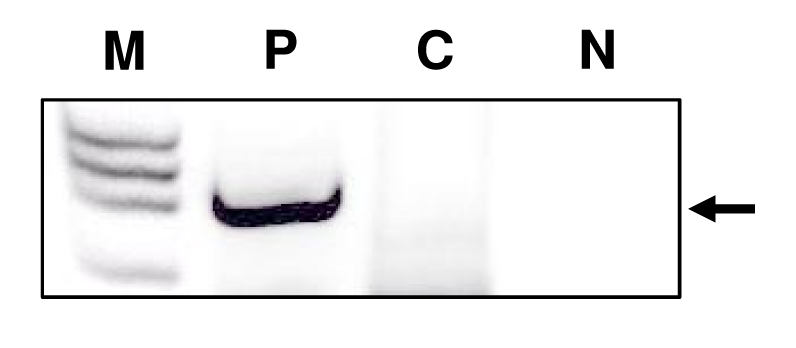

Supplement: S3 Fig — The arrow denotes the duplication-specific product. M: marker; phiX174 RF DNA/Hae III Fragments, P: patient 32, C: control, N: negative control, no DNA added. (TIF) [file pone.0181791.s003.tif]

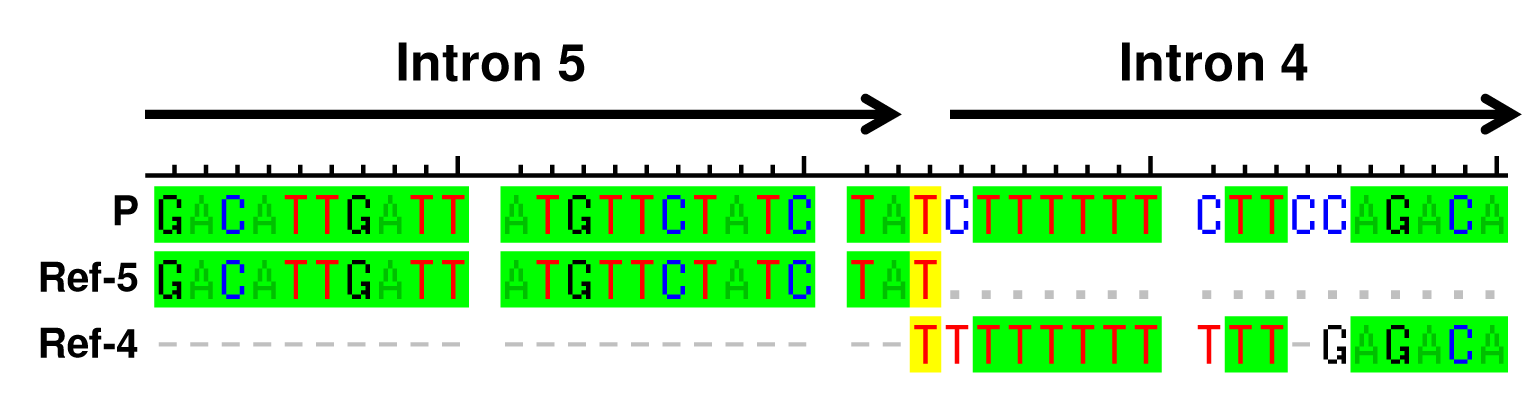

Supplement: S4 Fig — P: sequence around the breakpoint of the patient, Ref-4 and Ref-5: reference sequences of a part of introns 4 and 5, respectively. (TIF) [file pone.0181791.s004.tif]

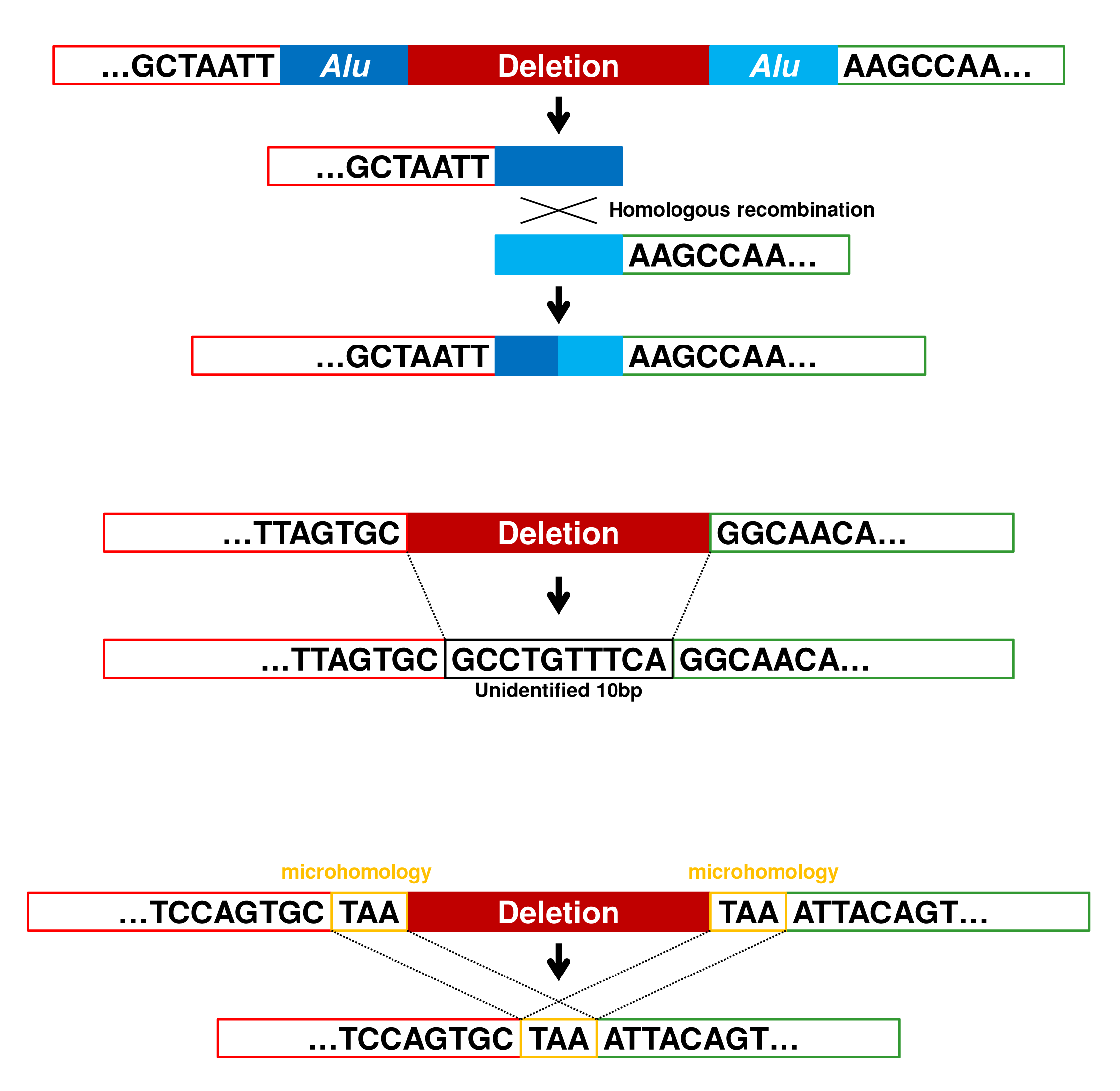

Supplement: S5 Fig — The size of each component doesn’t reflect the original proportion. (Upper) Two blue boxes denote Alu repeats flanking the deletion in patient 21. In the sequence of both proximal and distal Alu repeats, 118 of 126 bp (93.7%) are homologous, which likely induced the non-allelic homologous recombination (NAHR), resulting in the deletion. (Middle) There was no homology around the BP in patient 22 and the deletion might have been induced incidentally, while 10-bp sequences of unknown origin were observed at the BP. (Lower) Two yellow boxes denote 3-bp microhomologies, which likely induced the deletion in patient 23. (TIF) [file pone.0181791.s005.tif]

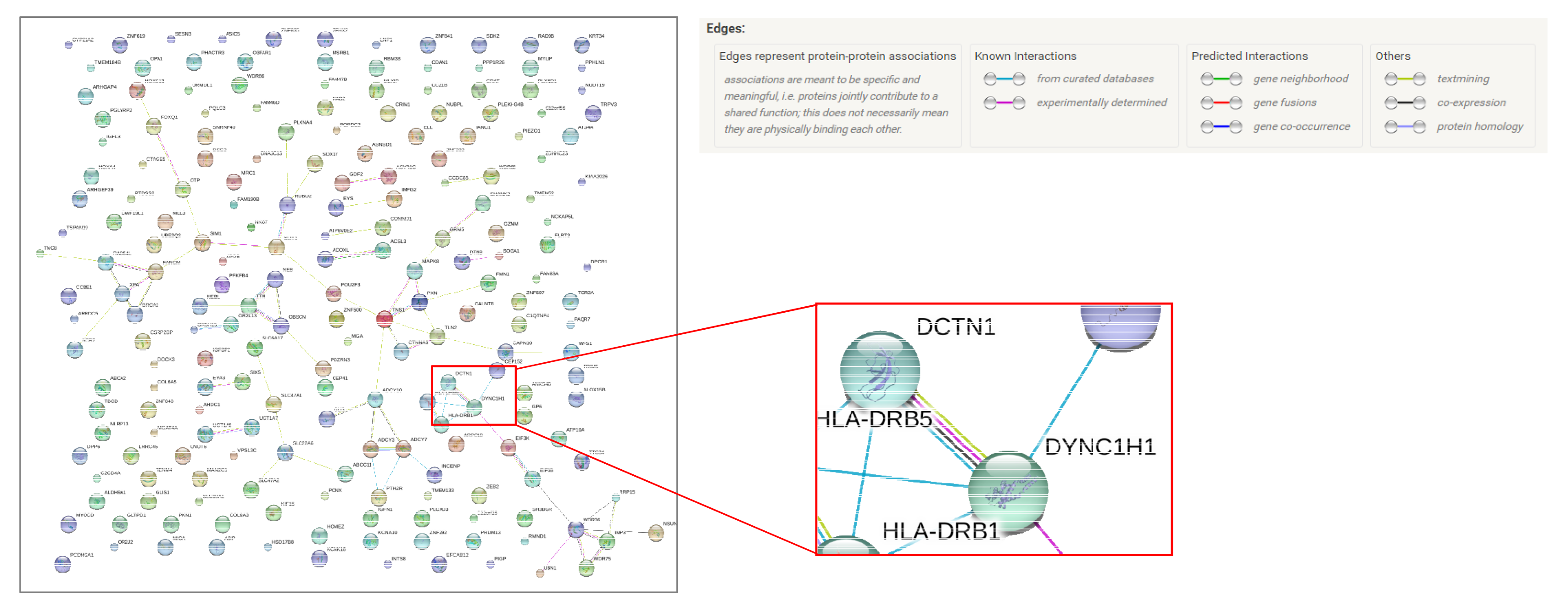

Supplement: S6 Fig — The WES analysis identified mutations including nonsense, splice-site, frameshift, and missense mutations, in those genes in patient 37. The enlarged panel shows a direct interaction between DYNC1H1 and DCTN1. (TIF) [file pone.0181791.s006.tif]

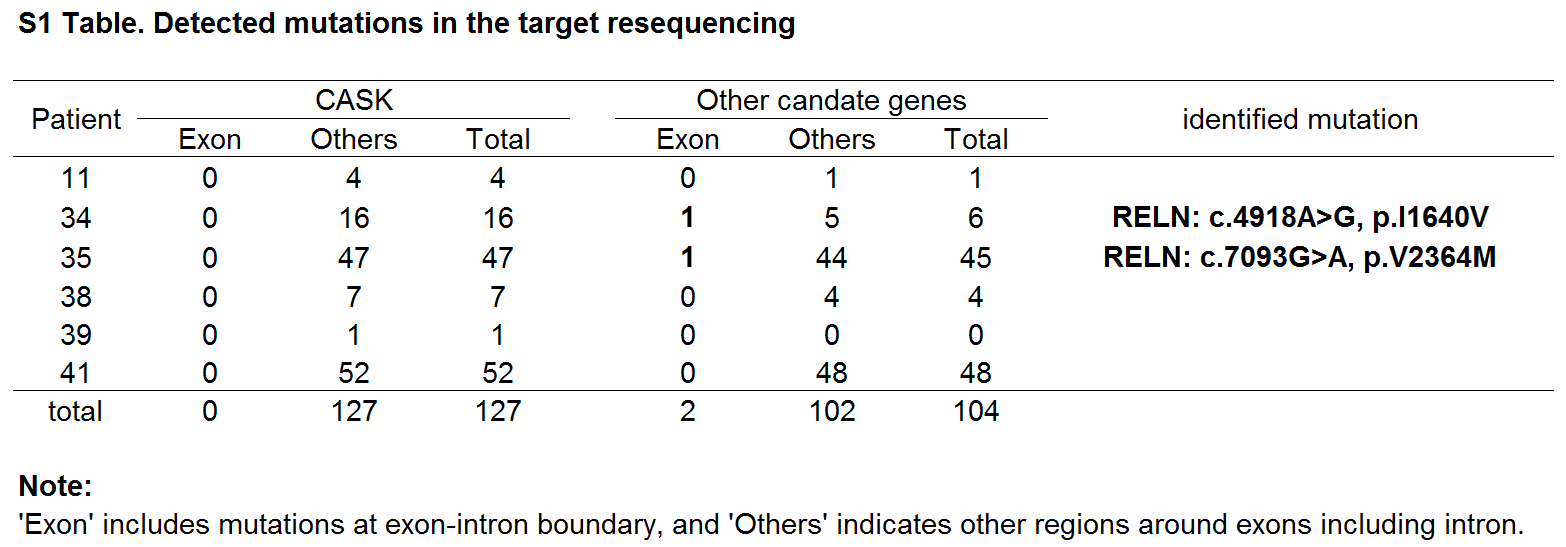

Supplement: S1 Table — (TIF) [file pone.0181791.s007.tif]

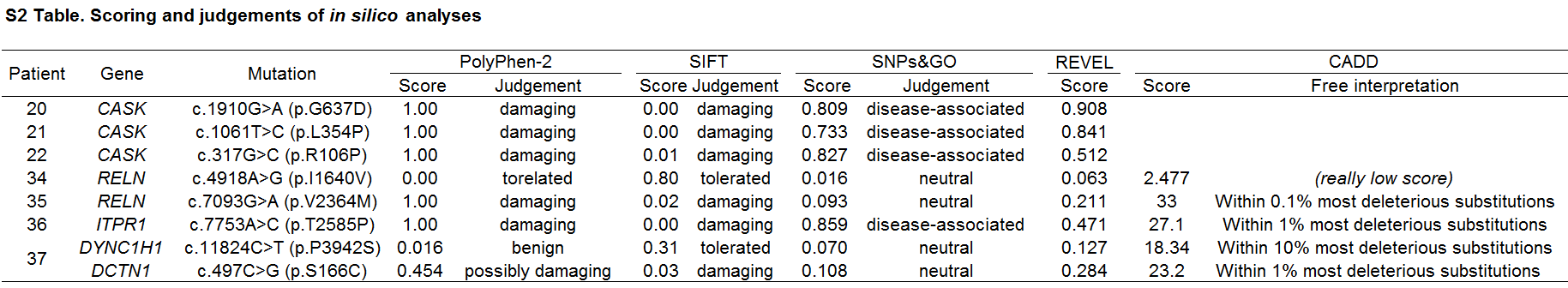

Supplement: S2 Table — (TIF) [file pone.0181791.s008.tif]

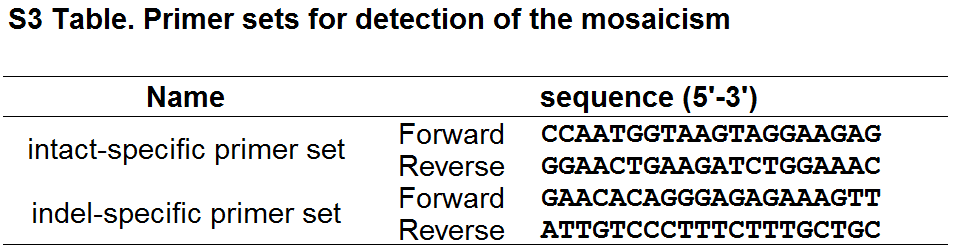

Supplement: S3 Table — (TIF) [file pone.0181791.s009.tif]
